# Editorial: Recent advances in medical radiation technology

**DOI:** 10.3389/fchem.2024.1360379

**Published:** 2024-01-11

**Authors:** Samir Acherar

**Affiliations:** Université de Lorraine, Centre National de Recherche Scientifique (CNRS), Laboratoire de Chimie Physique Macromoléculaire (LCPM), Nancy, France

**Keywords:** non-ionizing radiation, ionizing radiation, diagnosis applications, therapeutic applications, theranostic applications, radiation medicine

Personalized medicine and early diagnosis are being increasingly used in hospitals and clinical settings and are part of the most promising tools in current healthcare trends. In particular, many pharmaceutical companies develop and offer personalized healthcare solutions. Advances in medical radiation technology have profoundly affected medicine. Continued progress in this area for over a century had led to the emergence of innovations medical diagnosis and therapy that have completely reconfigured the identification and imaging of pathologies and the treatment of disease.

There are two types of radiation, namely, ionizing and non-ionizing radiation. Non-ionizing radiation comprises the electromagnetic spectrum portion, such as microwave (MW), infrared (IR), ultra-violet (UV) and visible light, for which the energy is sufficient only to enable excitation, but not enough to cause ionization. Ionizing radiation consists of high-energy radiation including electromagnetic radiation (gamma rays and X-rays) and particulate radiation (i.e., α, β and neutron particles from radionuclides).

For more than a century, the enthusiasm for the medical use of ionizing and non-ionizing radiation therapy, either alone or in combination with other modalities (e.g., chemotherapy, immunotherapy…) has continued to grow. Furthermore, these various radiation types have also been highly investigated for their usefulness in diagnosis.

As regards the ionizing radiation, their medical uses include, but is not limited to, nuclear medicine where radionuclides are used for therapy (e.g., radionuclide therapy) and diagnosis examinations (e.g., positron emission tomography (PET) and single-photon emission computed tomography (SPECT) imaging), but also X-ray radiography for similar diagnostic purposes.

Non-ionizing radiation is used in medicine as a powerful tool for the diagnosis (*e.g.*, magnetic resonance imaging (MRI), UV scans, medical infrared thermography (MIT), endoscopy...), and also for earlier diagnosis of diseases, especially tumors. There are also used for the treatment (e.g., photodynamic therapy (PDT), antimicrobial PDT (aPDT), photothermal therapy (PTT), radiotherapy (RT)...), diagnosis (e.g., photoacoustic imaging…) of various types of diseases, and for tissue regeneration.

This current Research Topic, comprising one Study Protocol and three Original Research articles, is focused on the recent advances in medical radiation technology, which covered topics ranging from radiation therapy to radiation diagnosis or with a combination of both (i.e., theranostic approach such as image-guided radiation therapy) using ionizing and non-ionizing radiation.

Population aging is an irreversible global trend with economic and socio-political consequences. One of the most undermining outcomes of aging in the elderly is the cognitive decline leading to dementia and neurodegenerative disorders. Alzheimer’s disease (AD) is the most common neurodegenerative dementia disorder impacting approximately 40 million patients worldwide (Naqvi, 2017). Without a cure or an effective therapy, the socio-economic impacts of AD upon the society are just going to intensify. In attempting to limit these impacts, Agger et al. described an interesting planned protocol study, which is a randomized, double-blinded, placebo-controlled clinical trial (ALZLIGHT, NCT05260177), to further the understanding of the use of flickering light stimulation as a treatment for AD. 62 patients with mild-to-moderate AD have participated to this clinical trial (half of those are treated and the others received placebo). The main advance of this study is the use of a novel invisible spectral flicker (ISF) device (i.e., 40 Hz non-invasive light therapy system) that switches between two light colors, as compared to previous studies that have used white light flickering on/off (Ismail et al., 2018; Hajos et al., 2020; Chan et al., 2021; Cimenser et al., 2021). A better placebo control is achieved, because the continuous white light delivered as placebo by the sham device is apparently perceptually indistinguable from the two light color profiles achieving a 40 Hz flicker.

According to the fact sheet from the World Health Organization (WHO) on lung cancer (World Health Organization, 2023), lung cancer is the leading cause of cancer death worldwide with an estimated 1.8 million deaths (18%) in 2020. Thoracic RT has been a treatment for locally advanced lung cancer, in combination or not with chemotherapy. Even is the treatment outcome has improved over the last decades, thoracic RT can inflict severe injuries, especially radiation pneumonitis (RP) for around 5%–30% of patients, which is the primary dose-limiting toxicity associated with radiotherapy. In this context, Zheng et al. proposed an interesting way to mitigate the incidence of thoracic RT on Chinese patients. The idea was based on the concurrent use of renin-angiotensin system inhibitors (RASi) that sought to limit the effects of RP. This study enrolled 320 patients (mean age of patients was 65 years) with various diagnosis, stage and treatment of lung cancer who received thoracic RT. These results suggested that the oral administration of RASi can mitigate the incidence of ≥ grade 2 RP in patients with lung cancer undergoing thoracic RT.

The poor prognosis of most cancers can be explained by their late diagnosis. This delayed diagnosis of cancers in a late stage often leads to the impossibility of doing surgery, or the already presence of metastatic cells. Therefore, various studies have been published or are underway to identify new biomarkers which could confirm the presence of cancers at an earlier stage, or which could better target cancer cells specifically and spare healthy tissues. In this way, Chen et al. were interested in 6-phosphofructo-2-kinase/fructose-2,6-bisphosphatase 3 (PFKFB3), which is a glycolysis regulatory enzyme overexpressed in a variety of human solid tumors, such as lung, breast, ovarian, pancreatic and colon cancers (Kotowski et al., 2021). Among the various studied selective PFKFB3 inhibitors, an aminoquinoxaline derivative gave the best IC_50_ value of 2 nM. This derivative was covalently linked to a gallium chelator, DOTA, and rabiolabeled to produce the PET tracer **Ga-5**. Molecular docking and *in vitro*/*in vivo* studies revealed the potential interest of **Ga-5** as selective PFKFB3-targeted PET tracer.

The construction of nanotheranostic agents (i.e., multi-functional nano-platforms combining diagnosis and therapy) remains a tough challenge to attain the goal of precise medicine (Kim et al., 2019). Among the various technologies made available to researchers, ionizing and non-ionizing radiation-based technologies are an essential tool for diagnosis and treatment purposes. Therefore, Wu et al. prepared novel carbon nitride-Rose Bengal nanoparticles (CN-RB NPs) using graphite-phase carbon nitride (g-C_3_N_4_) as skeleton. The authors used these CN-RB NPs for photothermal/radiation therapy under the guidance of near-infrared II (NIR-II) photoacoustic imaging to treat breast cancer. *In vitro* and *in vivo* studies revealed the significant photothermal effect of CN-RB NPs under 1,064 nm laser irradiation, along with an obvious *in vivo* photoacoustic signal for imaging. Interestingly, introduction of iodine element resulted in enhancing the radiation therapy capability of CN-RB NPs. *In vivo* studies showed also an obvious inhibitory effect on breast cancer tumors under irradiation of a 1,064 nm laser and/or X-ray highlighting the potential use of the CN-RB NPs to achieve high therapeutic effects and deep treatment.

In conclusion, this present Frontiers Research Topic offers an overview of the interest in medical radiation technology in both research and clinical purposes.
